# Evaluation and Verification of Fast Computational Simulations of Stent-Graft Deployment in Endovascular Aneurysmal Repair

**DOI:** 10.3389/fmedt.2021.704806

**Published:** 2021-07-20

**Authors:** Aymeric Pionteck, Baptiste Pierrat, Sébastien Gorges, Jean-Noël Albertini, Stéphane Avril

**Affiliations:** ^1^Mines Saint-Etienne, Univ Lyon, Univ Jean Monnet, INSERM, U1059 Sainbiose, Centre CIS, Saint-Etienne, France; ^2^THALES, Microwave & Imaging Sub-Systems, Moirans, France; ^3^INSERM, U1059 Sainbiose and University Hospital of Saint-Etienne, Univ Jean Monnet, Saint-Etienne, France

**Keywords:** aneurysm, computer assisted surgery, stent graft, endovascular aneurysm repair, reduced order model (ROM)

## Abstract

Fenestrated Endovascular Aortic Repair, also known as FEVAR, is a minimally invasive procedure that allows surgeons to repair the aorta while still preserving blood flow to kidneys and other critical organs. Given the high complexity of FEVAR, there is a pressing need to develop numerical tools that can assist practitioners at the preoperative planning stage and during the intervention. The aim of the present study is to introduce and to assess an assistance solution named Fast Method for Virtual Stent-graft Deployment for computer assisted FEVAR. This solution, which relies on virtual reality, is based on a single intraoperative X-ray image. It is a hybrid method that includes the use of intraoperative images and a simplified mechanical model based on corotational beam elements. The method was verified on a phantom and validated on three clinical cases, including a case with fenestrations. More specifically, we quantified the errors induced by the different simplifications of the mechanical model, related to fabric simulation and aortic wall mechanical properties. Overall, all errors for both stent and fenestration positioning were less than 5 mm, making this method compatible with clinical expectations. More specifically, the errors related to fenestration positioning were less than 3 mm. Although requiring further validation with a higher number of test cases, our method could achieve an accuracy compatible with clinical specifications within limited calculation time, which is promising for future implementation in a clinical context.

## Introduction

Abdominal aortic aneurysms (AAAs) are pathological dilations of the aorta with diameters larger than 50% of the normal physiological size. AAAs are commonly asymptomatic but they need to be treated through surgical interventions when they reach critical conditions. AAAs affect an increasing number of people, especially due to the aging and increasing life expectancy of the world's population. AAA rupture can occur when they reach critical conditions, causing death in 90% of cases. AAA rupture is responsible for 10,000 deaths in the United States every year (1). Endovascular aneurysm repair (EVAR) and fenestrated EVAR (FEVAR) are common mini-invasive treatments for AAA. EVAR consists in deploying a stent-graft in the patient's aorta, using an incision in the groin and access through the femoral arteries to reach the AAA. Within FEVAR, fenestrations and scallops must be minutely positioned in front of critical branch arteries for preserving the blood flow toward kidneys and other organs. The challenge is to ensure an accurate alignment between the stent-graft fenestrations and ostia. When the stent-graft is accurately positioned, catheterization of the branch arteries is facilitated and this was shown to significantly reduce the rate of postoperative complications such as thrombosis and embolization (2–4). However, positioning the stent-graft accurately is extremely challenging, especially for moderately experienced surgeons. Should difficulties occur, e.g., when juxtaposed ostia may divert the catheterization, the practitioner has to repeat fluoroscopy acquisitions with different viewing angles to mentally reconstruct the scene. This leads to an increase of the patient's irradiation and of the volume of injected contrast agent. These difficulties are amplified with the lack of available three-dimensional information about the current geometry and positions of the artery and of the stent-graft. Modern hybrid rooms can provide accurate 3D fusion, allowing to visualize the aorta and the iliac arteries in real-time during an EVAR intervention. Moreover, rotational fluoroscopy acquisitions enable to verify if stent grafts are accurately deployed with different view angles (5, 6). However, a large number of operating rooms are still equipped with simple mobile C-arms, which do not offer rotational fluoroscopy acquisitions.

Therefore, there is still a pressing need to develop virtual reality (VR) technologies for EVAR assistance, which should satisfy two major goals:

(1) they should enable 3D reconstructions of the aortic geometry and update this geometry when it is deformed by guidewires during stent-graft insertion.
(2) they should make predictions of FEVAR outcomes such as simulating stent-graft deployment for different interventional scenarios.

Further specifications need also to be satisfied by these VR technologies:

(1) the total computation time must be compatible with clinical expectations, which does not exceed a few minutes.
(2) they must not disturb the operating flow and cause any additional irradiations or contrast agent injections. Then, a minimal number of images should be required by the tool.

The research community has been very active in proposing VR solutions to these goals and specifications. Regarding the first goal, different approaches are currently available: pre-computing deformation during pre-operative planning (7, 8), using two fluoroscopic images (9–11), using a single fluoroscopic image coupled to length regularization (12), or using graph-matching (13). We recently proposed a finite element model of the aorta with geometric constraints extracted from intraoperative images (14). Regarding the second goal, many studies have modeled stents (15–19) or stent graft (20–22) deployment in patient-specific geometries using the finite-element method. However, these models were generally computationally expensive, requiring several hours of simulation, and they did not take intraoperative data into account.

More recent studies have focused on developing reduced order models for stent-graft deployment based on constrained deformable simple models (23–25), mass-spring models (26, 27) or active contours (28). Although faster, these methods did not integrate intraoperative data. Numerical simulations integrating intraoperative images remain scarce. Demirci et al. (29) proposed to recover the position of stents by geometrical reconstruction from a single intraoperative view. More recently, a real-time framework based on a single 2D fluoroscopic image and the positions of radiopaque markers were used to generate the 3D shape of a fenestrated stent graft (30, 31). Despite the lower computational time, interventions with complex aortic geometries or complex stent-grafts remained too challenging for this framework. The accuracy was also a weakness of these approaches, compared to standard finite-element models.

Herein, we propose a new VR solution for computer assisted (F)EVAR based on a single X-ray image, named Fast Method for Virtual Stent-graft Deployment (FMVSD). This hybrid method simultaneously includes the use of intraoperative data and of a simplified mechanical model, making a very good tradeoff between precision and computation time, which is compatible with clinical expectations. Our solution relies on a single intraoperative X-ray image that can be provided by a standard mobile C-arm and that can handle fenestrations positioning.

The objective of this paper is to assess and verify the performances of the FMVSD solution. In the methods section, we introduce the details of the algorithm, the different assumptions and the assessment method. In the results section, we first show qualitative verifications of the FMVSD and then provide the quantitative assessment results. The interest of the approach and its limitations are further discussed in the discussion section.

## Methods

### Interventional Scenario

In this subsection, we describe the different steps of an EVAR intervention that we wish to assist with our method, and the main assumptions made for the VR representation. During the pre-operative phase of EVAR, a 3D CT scan of the aorta is acquired for the planning purpose. This 3D scan can used to reconstruct the reference geometry of the aorta and of the other blood vessels. During standard EVAR, the practitioner makes a small incision in the femoral artery, inserts a guidewire and then inserts a launcher, which is a sheath containing the stent-graft. The stent-graft is usually composed of several parts, which are inserted sequentially. In this study, we focus on the insertion and deployment of the main body of the stent-graft. When the launcher reaches the AAA, the stent-graft is progressively deployed. In FEVAR, the fenestrations of the stent-graft must be positioned precisely in front of the renal and other branch ostia to allow their catheterization. The ostia are usually about 5-7mm diameter. Throughout the surgery, we assume that the practitioner is guided by an X-ray imaging system mounted on a mobile C-arm that provides 2D images. The projection matrix associated with the mobile C-Arm is assumed to be known, through calibration or complementary devices (32).

### Description of the FMVSD Algorithm

**Figure 1:**
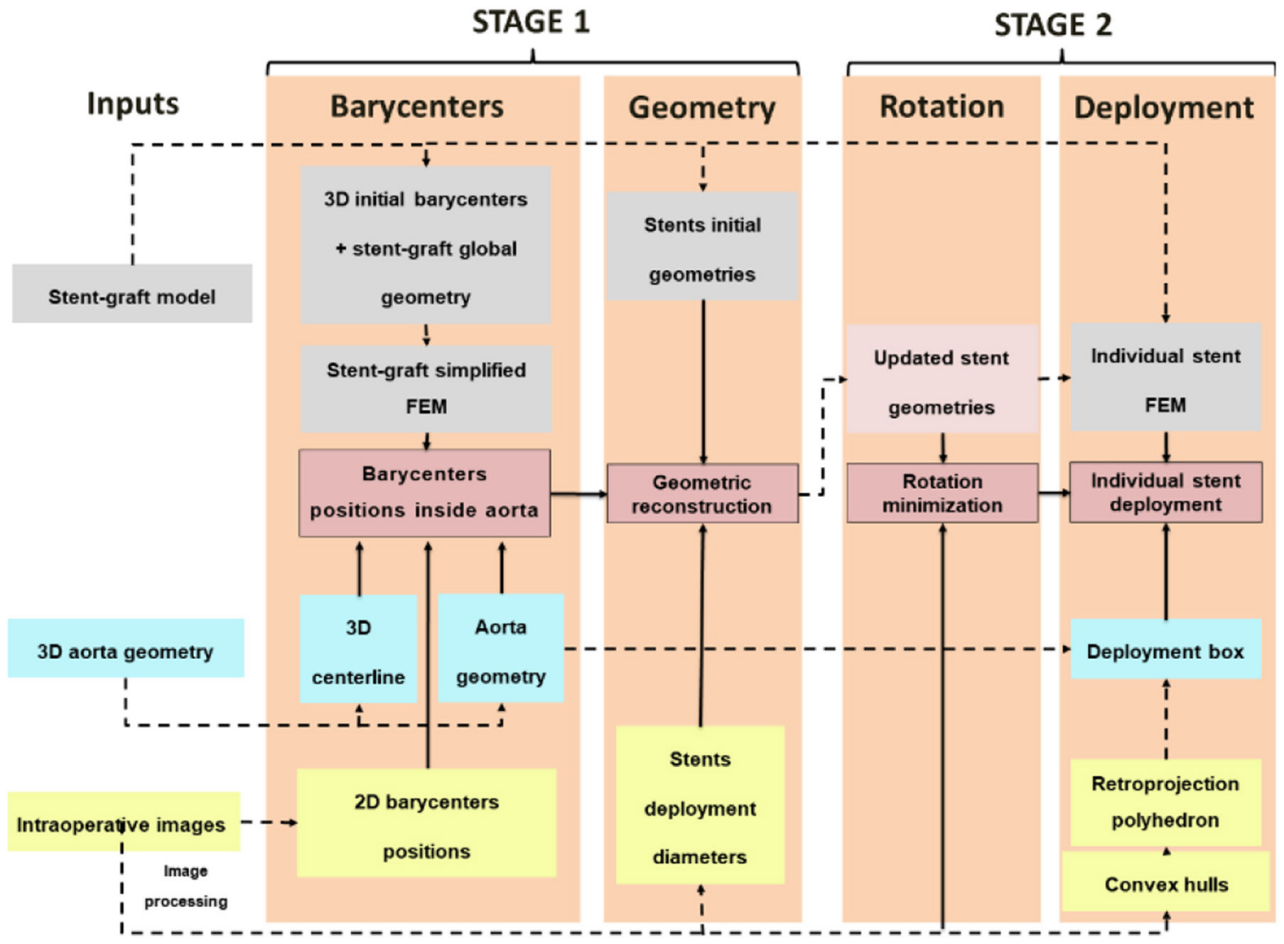
Summary of the algorithm of FMVSD.

The FMVSD algorithm, which was introduced in a previous study (33), is shown in Figure 1. In brief, the algorithm is divided into two Stages and requires three inputs. Stage 1 provides a first approximation of the global deployment of the stent-graft in a short computation time. During the first step of Stage 1, barycenters of each stent are positioned in the three dimensional space using a finite element model (FEM) of the stent-graft in the aorta. Then, the stents are geometrically reconstructed around the new position of their barycenter during the second step of Stage 1. Stage 2 improves the outputs of Stage 1 at the cost of a higher computation time. The first step of Stage 2 consists in recovering the rotation of the stent around its main axis through a minimization loop. The second step of Stage 2 consists in deploying each stent individually using individual stent FEM. First, we will describe the inputs of the algorithm before presenting Stages 1 and 2 in details.

The FMVSD method requires three inputs:

(1) the intraoperative 3D geometry of the aorta,
(2) the stent-graft FEM,
(3) and the X-ray intraoperative image of the stent-graft (target image).

To obtain the intraoperative geometry of the aorta, the preoperative geometry acquired before the intervention must be registered to match the intraoperative aorta geometry deformed by the guidewires and launcher. Centerlines were extracted using the Voronoi diagram method implemented in the VMTK library (34). We proposed previously a non-rigid registration method based on intraoperative images of the aorta and a FEM of the aortic centerline (14). The updated geometry was converted into a triangular surface mesh easily implementable in an FEM. Assuming that non-rigid registration was previously performed, the 3D geometry of the aorta perfectly matches the real geometry. The stent-graft models were then obtained from manufacturer specifications and discretized into finite-elements using a dedicated Matlab® routine. The stent-graft intraoperative target images were acquired during the surgery through mobile C-arm. As all simulations were constrained and guided by intraoperative imaging, relevant information were extracted from the target image. To isolate the contour of the stents, we applied a combination of Frangi filters and masks to the image (29). Then, we extracted the convex hull of each stent. The two-dimensional pixel coordinates of the barycenter of each stent were simply obtained from the convex hull. We also measured apparent deployment diameters.

**Figure 2:**
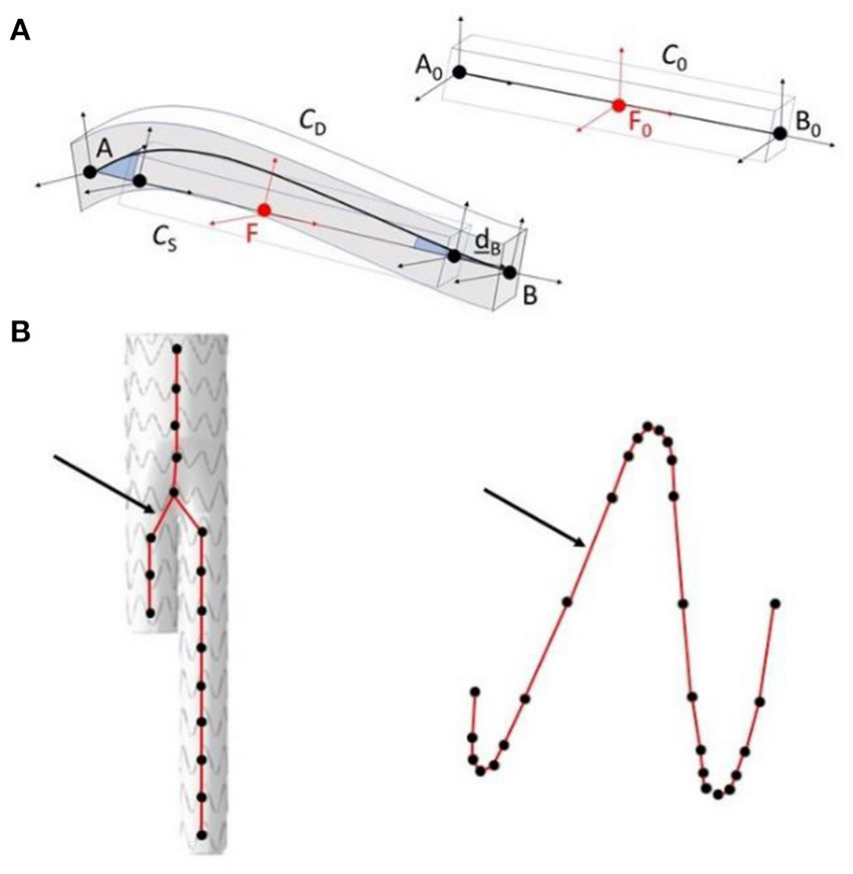
Schematic representation of the corotational approach **(A)**; application in the method, in step 1 of stage 1 modeling of the stent-graft centerline (on the left), in step 2 of stage 2 modeling of the stent struts (on the right) **(B)**. The arrows show corotational beam elements between connecting nodes.

The main algorithm was divided into four steps (33). The first two steps were combined into a single stage, named Stage 1. As it had a marginal computational cost, this Stage could be run in less than 20s. However, it was not accurate enough. Thus, the output of Stage 1 was used as input for Stage 2 to improve the precision of simulations. In the first step of Stage 1, a simplified FEM version of the stent-graft, reduced to its centerline, was positioned in the aorta geometry. The FEM was composed of corotational beam elements, each beam representing a stent. The corotational approach, based on classical linear finite elements, is very versatile and allows simulating large deformations (Figure 2). A floating coordinate system **F** follows the deformed element, so that the overall movement in the deformed **C**_D_ state can be decomposed into a large rigid body motion from the reference configuration **C**_0_ to the so-called floating or phantom configuration **C**_S_, times a small local deformation from **C**_S_ to **C**_D_. The underlined symbols represent variables expressed in the floating reference basis **F**. A global tangent stiffness ***Ke*** and a global force vector **fe** are derived for each element **e**, given its local matrix ***K***, its local force **f** and the rigid body motion of **F** in **C**_0_ to **F** in **C**_S_. At each time step, the position and rotation of **F** are updated. Beam elements were cylindrical, and each node had 6 degrees of freedom, 3 translations and 3 rotations. Each beam element was associated to a 12 x 12 elementary stiffness matrix **K** calculated in local coordinates that related angular and spatial position of each end of a beam element to the forces and torques applied to them (Equation 1):


(1) $$\begin{array}{l} \underline{K}=\frac{E}{l}\left[\begin{array}{llllllllllll} A & \; & \; & \; & \; & \; & \; & \; & \; & \; & \; & \;\\ 0 & \frac{12Iz}{l^{2}\left(1+\Phi y\right)} & \; & \; & \; & \; & \; & \; & \; & \; & \; & \;\\ 0 & 0 & \frac{12Iy}{l^{2}\left(1+\Phi z\right)} & \; & \; & \; & \; & \; & \; & \; & \;\\ 0 & 0 & 0 & \frac{GJ}{E} & \; & \; & \; & \; & \; & Symmetric & \; & \;\\ 0 & 0 & -\frac{6Iy}{l\left(1+\Phi z\right)} & 0 & \frac{\left(4+\Phi z\right)Iy}{1+\Phi z} & \; & \; & \; & \; & \; & \; & \;\\ 0 & \frac{6Iz}{l\left(1+\Phi y\right)} & 0 & 0 & 0 & \frac{\left(4+\Phi y\right)Iz}{1+\Phi y} & \; & \; & \; & \; & \; & \;\\ -A & 0 & 0 & 0 & 0 & 0 & A & \; & \; & \; & \; & \;\\ 0 & -\frac{12Iz}{l^{2}\left(1+\Phi y\right)} & 0 & 0 & 0 & \frac{-6Iz}{l\left(1+\Phi y\right)} & 0 & \frac{12Iz}{l^{2}\left(1+\Phi y\right)} & \; & \; & \; & \;\\ 0 & 0 & -\frac{12Iy}{l^{2}\left(1+\Phi z\right)} & 0 & \frac{6Iy}{l\left(1+\Phi z\right)} & 0 & 0 & 0 & \frac{12Iy}{l^{2}\left(1+\Phi z\right)} & \; & \; & \;\\ 0 & 0 & 0 & \frac{-GJ}{l} & 0 & 0 & 0 & 0 & 0 & \frac{GJ}{E} & \; & \;\\ 0 & 0 & -\frac{6Iy}{l\left(1+\Phi z\right)} & 0 & \frac{\left(2-\Phi z\right)Iy}{1+\Phi z} & 0 & 0 & 0 & \frac{6Iy}{l\left(1+\Phi z\right)} & 0 & \frac{\left(4+\Phi z\right)Iy}{1+\Phi z} & \;\\ 0 & \frac{6Iz}{l\left(1+\Phi y\right)} & 0 & 0 & 0 & \frac{\left(2-\Phi y\right)Iz}{1+\Phi y} & 0 & -\frac{6Iz}{l\left(1+\Phi y\right)} & 0 & 0 & 0 & \frac{\left(4+\Phi y\right)Iz}{1+\Phi y} \end{array}\right] \end{array}$$

with **G** = $\frac{\text{E}}{2\left(1+\text{v}\right)}$, where E is the Young's modulus and ν the Poisson's ratio; **I**_y_ and **I**_z_ are cross-section moments of inertia; **A** is the cross-sectional area of the beam, **l** its length; **ϕ**_y_ and **ϕ**_z_ represent shear deformation parameters and are defined as **ϕ**_y_ = $\frac{12\text{EIz}}{\text{GAsy}{\text{l}}^{2}}$ and **ϕ**_z_ = $\frac{12\text{EIy}}{\text{GAsy}{\text{l}}^{2}}$ with **A**_sy_ and **A**_sz_ the shear area in the y and z directions. In the first step of Stage 1, **E** = 10MPa and **ν** = 0.3. These values were adjusted by trial and error in order to ensure the robustness and stability of the model. High Young's modulus values led to instability and high potential energy as they overconstrained the model. On the other hand, low Young's modulus resulted in rubber-like unrealistic configuration. These values were also adjusted by qualitatively comparing the simulations results with finite element simulations performed with Abaqus®.

Each beam was connected to its neighbors, constrained in translation but free in rotation to mimic the restriction of stent motion when sewn to the fabric. This approximation allowed to simulate the cohesive role of the fabric between the stents, while keeping a short computation time. The centerline model was initialized in its unconstrained state, completely deployed. Then, the stent-graft was constrained in 3D through geometric estimations, based on the 2D target image and our knowledge of the projection matrix associated with the imaging device. Finally, the model was released, and constrained by the arterial wall, until reaching equilibrium. During the second step of Stage 1, we geometrically reconstructed the stents around the centerline, based on the orientation of the beam elements, the new position of the 3D barycenters and the apparent 2D deployment diameters of the stents. The initial geometry of each stent, *i.e*. the metal structure, was first discretized into a set of points. Each point was defined as a vector $\vec{V}\;\left(V_{x},V_{y},V_{z}\right)$, which originated from the three-dimensional stent barycenter **B**_3D_ and was expressed in the stent local coordinate system. The local deployment diameter of each stent was therefore interpolated based on proximal and distal diameters, which were measured during the image processing step. New reconstructions vectors $\vec{V_{D}}$ were updated according to the measured deployment diameters and expressed in the global reference system. Finally, new positions of the stents were reconstructed based on $\vec{V_{D}}$ and new positions of the barycenters.

Following Stage 1, individual stent deployment suffered from a lack of accuracy and needed to be improved. Two refining steps were achieved during Stage 2. These supplemental steps required a slightly longer computation time (up to 6 min) but reached higher accuracy. Stage 2 combined two individual refining steps: rotation minimization of stents around their longitudinal axis and individual deployment of stents. The first step was performed within a minimization loop to match the projection of the 3D stent, reconstructed during Stage 1, with the 2D intraoperative target stent image. The value to be minimized was the average distance between each point of the reconstructed stent and its nearest target neighbor. The variable was the angle of rotation of the stent around its longitudinal axis, and a differential evolution algorithm was used to perform the minimization. The 3D model of the stent geometrically reconstructed at the end of the previous step was projected according to the projection parameters of the target image. *d* was the average distance between each point of the reconstructed stent and its nearest neighbor. In the case of axisymmetric stents, a loop was used to determine the proper rotation Φ ± *k*θ, where θ is the periodic angle separating two peaks of the Z-shape axisymmetric stent and *k* a real integer. Considering fenestrated stent-grafts, all stents with fenestrations or scallops had radiopaque markers to guide the positioning. The new *d*_*RM*_ deviation to be minimized was calculated by considering only the distance between the radiopaque markers. In this case, the proper rotation Φ was exact and did not depend on θ. Indeed, the position of fenestrations was asymmetrical.

In a clinical context, deployment of each stent can be simulated individually regarding surgeon appreciation. In this study, we simulated every stent deployment to fully evaluate the performance of the method. First, we extracted a rigid deployment box, defined as the Boolean intersection between the back-projection polyhedron of the 2D convex hull of the target stent and the aorta volume. This back-projection was performed based on our prior knowledge of the projection matrix associated with the mobile C-Arm. The rigid deployment box was used to constrain the deployment of individual stent FE models. Corotational Euler-Bernoulli beam elements with a linear elastic behavior (35) were used to model the stents struts. The mesh was inhomogeneous and was denser in the areas of high curvature, with a mesh size ranging from 2 to 0.1 mm. The stent model was initialized in its deployed configuration, its rotation Φ around its main axis corrected using the output of step 1, and pre-constrained to the diameter of the stent graft launcher before the simulation begins. The stent beams had a diameter of 0.125 mm and were made of 316L steel, which mechanical characteristics are summarized in (36). The deployment was calculated using the Project Chrono engine (37), with the Pardiso solver of the Intel® Math Kernel Library (MKL). After the first contact, the time step was reduced to ensure the stability of the model. Contacts were modeled using the penalty algorithm implemented in Project Chrono, the Smooth-Contact (SMC) modeling approach. Default parameters were used to define contact properties. SMC uses penalty (in a discrete element method regularizing the frictional contact forces with “imaginary” spring-dashpot systems at each contact), and as such objects in contact will have slight interpenetration and integration time-step will likely be small (38, 39). The simulations were performed on a computer with 4 CPUs, 3.40 GHz, 16 GB RAM in less than 6 min, without parallelization.

The final output was a set of meshes, one for each stent, composed of beam elements. The meshes were merged in a single file and then integrated with the surface mesh of the aortic wall geometry to facilitate rendering. In a clinical context, this rendering is superimposed on intraoperative images.

### Sources of Error

The objective of this paper is to evaluate the performance of the method. To be able to run the simulation in a time that is compatible with clinical expectations, a compromise between computation efficiency and model complexity was done. Consequently, we quantified the errors introduced by this compromise. In this section, the main assumptions of the method are listed and the resulting simulation errors are evaluated. In order to differentiate and analyze these different sources of error, two datasets were acquired with different conditions, in order to identify the sources of error that have the largest impact on the final simulation.

A stent-graft is composed of metallic stents tied to a waterproof fabric. The simulation of this membrane is really time consuming, especially considering contacts and self-contacts of the fabric, and was not implemented in the stent deployment step, during Stage 2. Actually, effects of fabric were considered in Stage 1 during stent-graft positioning in the aorta through the connections between the beam element of the centerline. The absence of the fabric surrounding the stents in individual stent deployment simulations was at the origin of the first Error Source (*ES*). Although needed to reduce computation time, the absence of fabric was a potentially critical source of error. Indeed, as the fabric is connecting the stents together, the deployment of a stent can influence the deployment of neighboring stents. This error was named *ES fabric*.

The aorta is a soft tissue, potentially deformable. During individual stent deployment (Stage 2), the aorta was assumed rigid. This simplification, also necessary to save computation time, neglected aortic wall deformations induced by stent deployment. Although probably moderate, the radial deployment force of the stent being low, this assumption could be a source of error, which was named *ES stiffness*.

### Data Acquisition

In order to isolate the different sources of error, two datasets were acquired. Each dataset was subject to different sources of error (Table 1). Thus, by comparing and cross-checking the results, it was possible to estimate the errors created by each source separately.

**Table 1:** Sources of error in each Dataset.

		**Sources of error**	
	**Description**	**ES fabric Fabric effect neglected**	**ES stiffness Local rigid aorta**
Dataset I	Rigid phantom	Present	Not present
Dataset II	Patient data – Aorta geometry from post-operative CT scan	Present	Present

**Figure 3:**
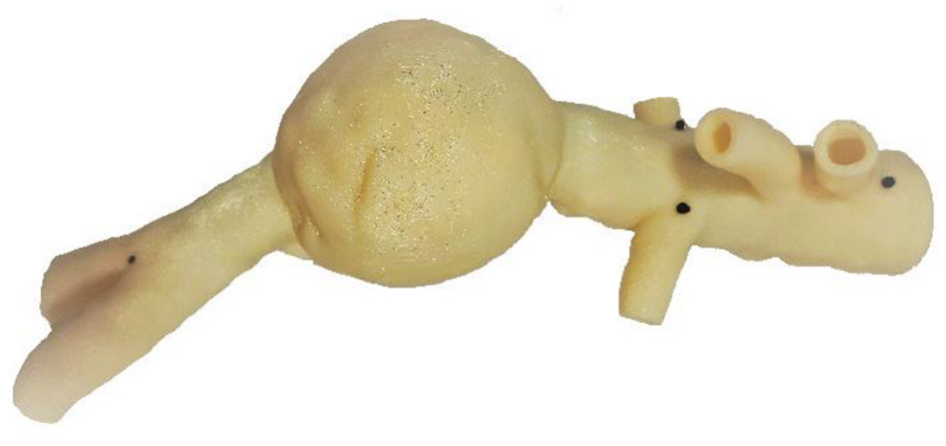
3D printed rigid phantom of an aorta for the validation of our simulations.

To generate Dataset I, we deployed a stent-graft in a 3D printed aorta phantom. This experiment was conducted in a hybrid room, which allowed us to acquire localized 2D images but also to perform a 3D rotational acquisition. The phantom was made of rigid plastic (Figure 3) and was based on a real aorta geometry segmented from EVAR patient. A rotational acquisition was performed to obtain the ground-truth 3D configuration of the stent-graft. Since the phantom was rigid, the simplification concerning the wall stiffness was verified. Consequently, the only source of error affecting the simulation was *ES fabric*.

**Figure 4:**
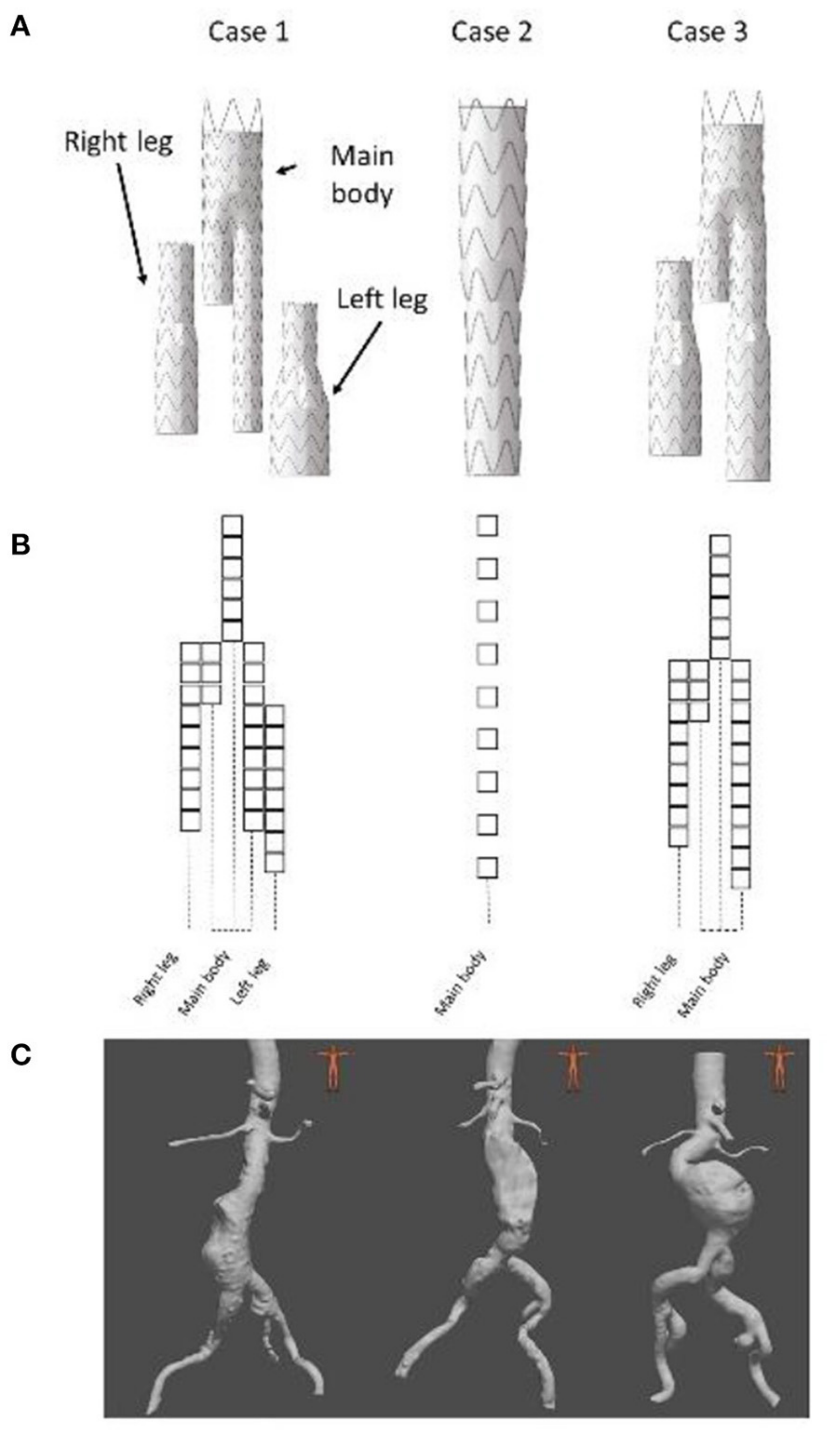
Presentation of the 3 clinical cases, stent graft models used during the EVAR **(A)**, stent graft corresponding schemes **(B)** and preoperative geometries **(C)**.

Since intraoperative data were not available, we generated Dataset II retrospectively using post-operative CT scans of three EVAR patients. All patients had given their consent for the use of their data. All data and images were acquired at the Saint-Etienne University Hospital under clinical conditions. Clinical data related to EVARs are summarized in Table 2 and Figure 4. The devices were manufactured by Medtronic (Santa Rosa, CA, USA). The maximum pixel size of the scanners was 0.9395 × 0.9395 mm^2^ and the maximum thickness of the slices was 2 mm. 2D target images were generated from the post-operative scanners. The geometry used as input for the FMVSD method was extracted from the pre-operative scanner, before stent graft deployment, and 3D registered on post-operative geometry. Therefore, the sources of errors were related to fabrics (*ES fabric*) and arterial wall stiffness (*ES stiffness*). Indeed, target images were extracted from real clinical data, the vessels were no longer rigid.

**Table 2:** Information about EVAR patients used for the validation of our simulations.

**Patient**	**1**	**2**	**3**
Sex	M	M	M
Age (years)	70	58	78
**Stent graft**			
Main body	ENBF-28-13-C-145-EE	Fenestrated stent graft	ENBF-28-20-C-170-EE
Right leg	ENLW-16-20-C-95-EE	–	ENLW-16-24-C-95-EE
Left leg	ENLW-16-28-C-80-EE	–	–
Proximal diameter (mm)	28	28	28
Distal diameter (mm)	13	13	20
Aneurysmal sac thrombus	Yes	Yes	No
Thrombus length (mm)	60	100	–
Maximal thrombus thickness (mm)	20	20	–

### Measurement and Evaluation of Errors

**Figure 5:**
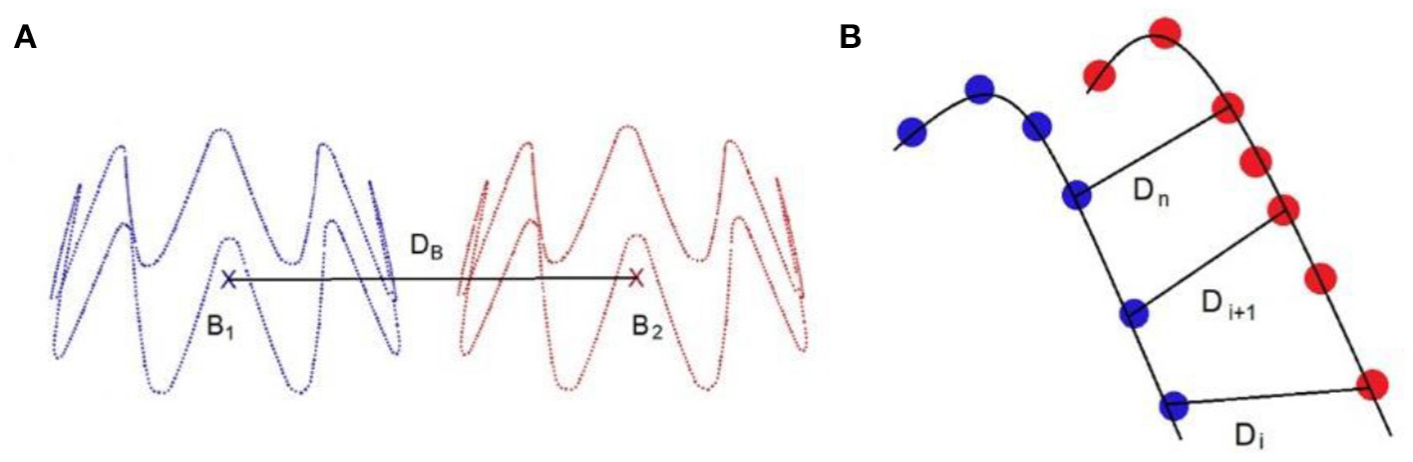
Distance measurement: D_B_ is the distance between the stents point clouds barycenters **(A)**, D_PC_ is the average distance between the closest neighbors of the point clouds of the target stent and the simulated sten, such as $D_{PC}=\overset{n}{\underset{i=1}{\sum }}D_{i}$ **(B)**.

Several measurements were used to evaluate the quality of the simulation (Figure 5). The first one is the **D**_B_ distance, which is the distance between the 3D barycenters of the target stent and the simulated stent. This distance was used to evaluate the quality of stent positioning within the artery. The second measurement was the **D**_PC_ distance, the average distance between the point clouds of the target stent and the simulated stent. It was defined as the average of the Euclidean distances separating a node of the simulated stent from its closest neighbor among the point clouds of the target stent. This distance allowed to assess at the same time the quality of stent positioning and deployment. In agreement with experienced clinicians and literature (40), simulation quality was qualified as *excellent* when **D** < 3 mm, *good* when **D** < 5 mm, and *insufficient* if **D** > 5 mm.

## Results

### Qualitative Results

**Figure 6:**
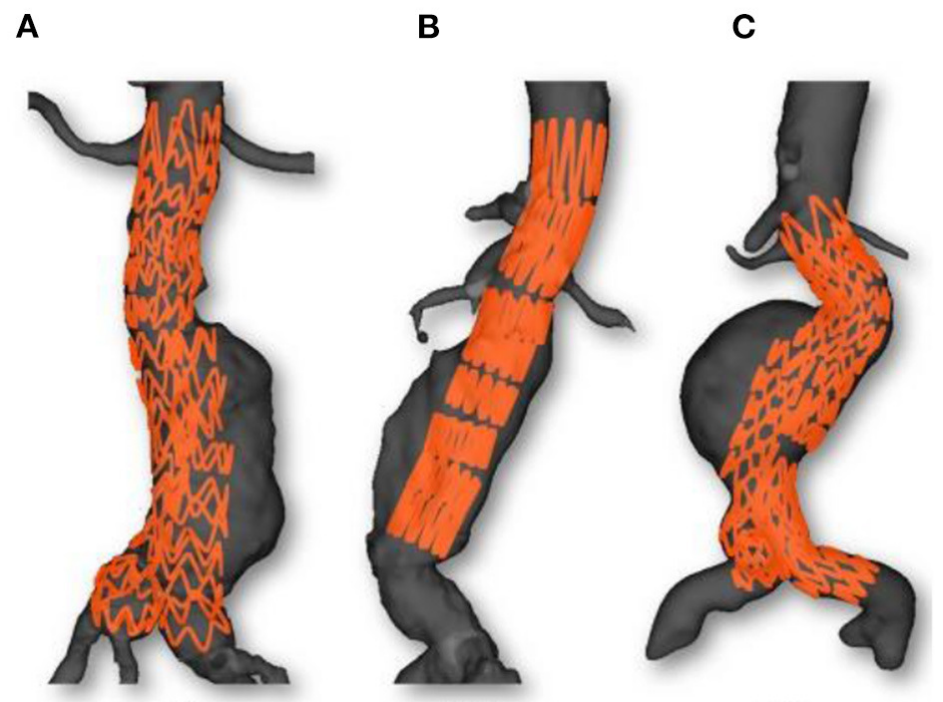
Simulations results: Case 1 **(A)**, Case 2 **(B)** and Case 3 **(C)**. Simulated stent grafts are represented inside the patient aorta.

**Figure 7:**
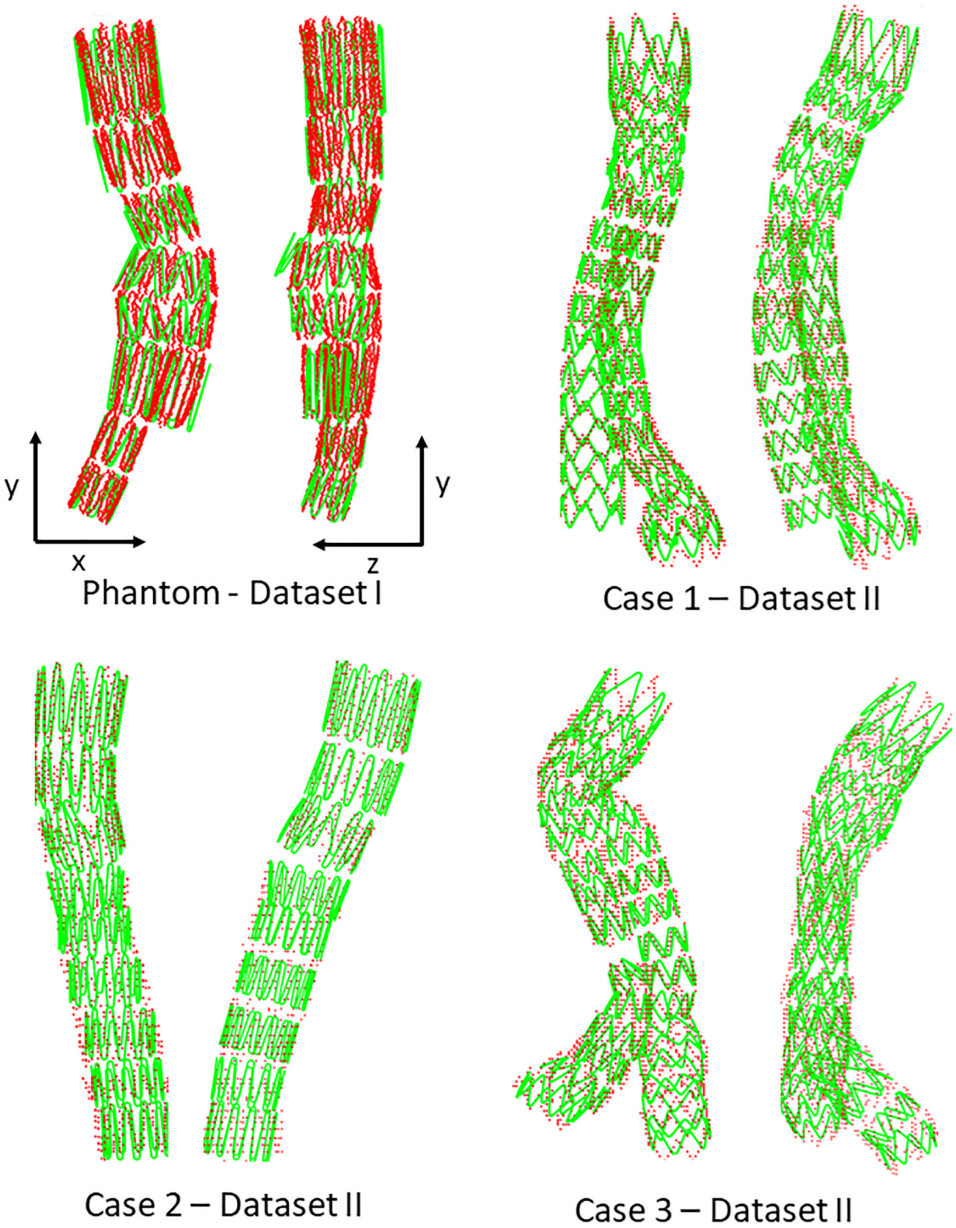
Comparison between simulation (green) and target stent graft (red), frontal view on the left, side view on the right. Reminder: Sources of error in Dataset I: *ES fabric*; Dataset II: *ES fabric* and *ES stiffness*.

Figures 6, 7 provide an overview of the simulation results. Figure 6 illustrates the results of the simulated stent-grafts within the arteries of the 3 patients. Figure 7 shows the superimposition of the target stents in red and the simulated stents in green. For each stent-graft a front and side view are shown. The front view [oriented according to (x,y)] corresponds to the image plane on which the simulation was based. The side view is oriented according to (y,z). The z-axis therefore corresponds to the projection axis.

**Figure 8:**
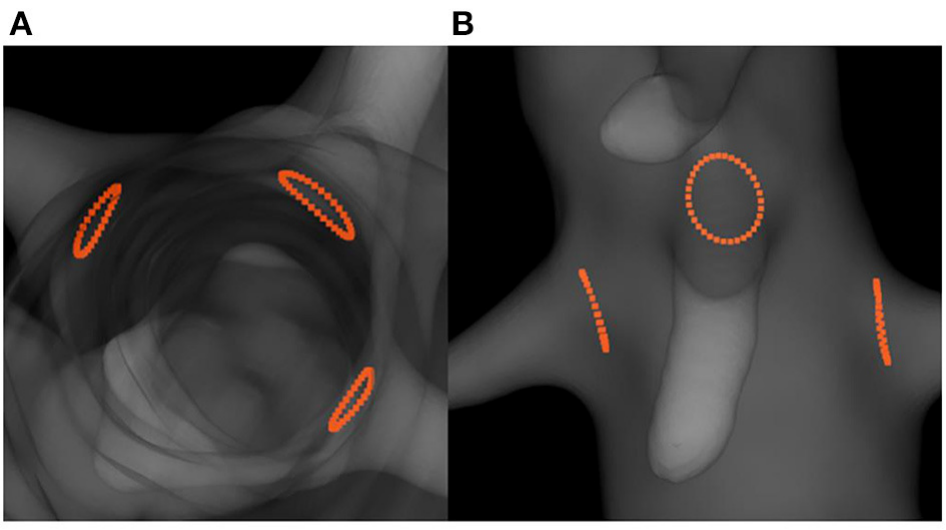
Simulation results showing the predicted positions of fenestrations with respect to the target ostia: top cross-section view **(A)** and frontal view **(B)**.

**Figure 9:**
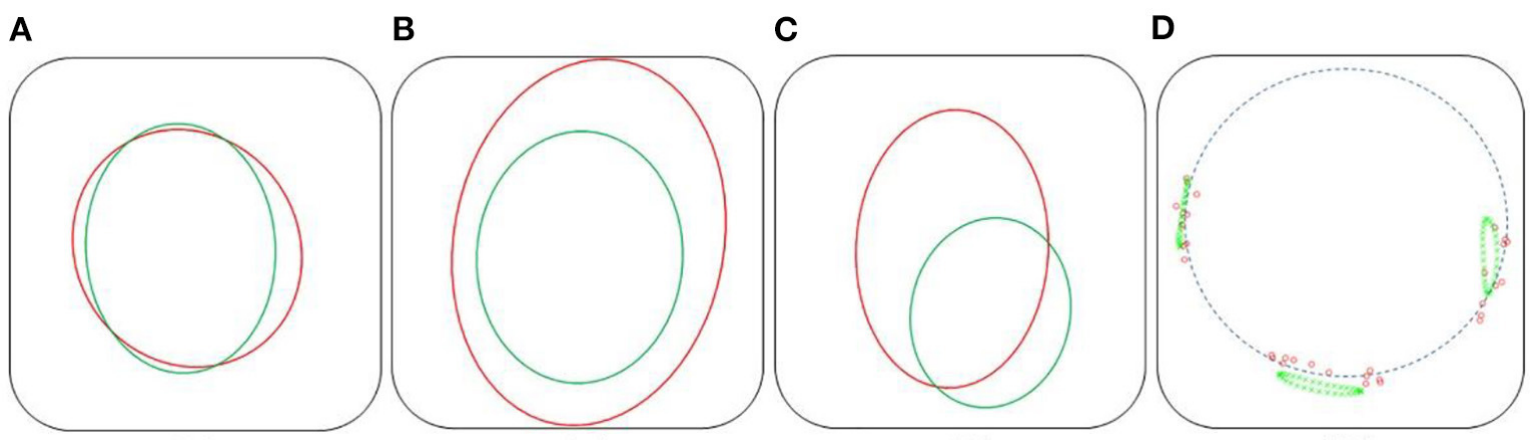
Comparison of fenestration positions. In red, the targets, in green the reconstructed fenestrations, dataset II. Frontal view: Right renal **(A)**, mesenteric **(B)**, and left renal **(C)** fenestrations. Top cross section view **(D)**, the dotted circle symbolizes the stent.

Positioning fenestrations is a critical step for the success of fenestrated EVAR. Figure 8 shows the simulated fenestrations inside the aorta. Figures 9A–C shows a comparison between the positions of target and simulated fenestrations, in frontal view. The target fenestrations appear in some cases larger than the simulated one. This was due to the artifacts generated by the radiopaque markers during X-ray imaging, which resulted in an apparent increase in marker size. Figure 9D also shows a comparison of fenestration position captured from above, which is equivalent to a cross section view of the stent. The stent is represented by the dotted circle.

### Quantitative Results

**Figure 10:**
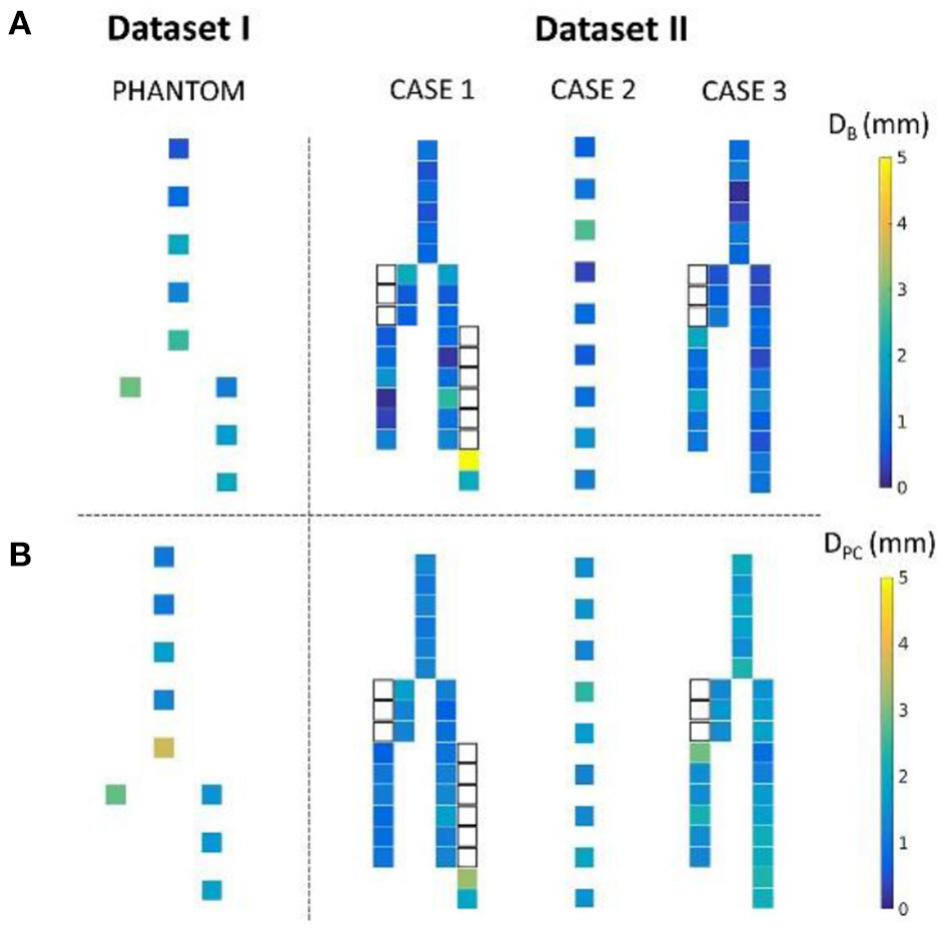
Distance map representing the distance D_B_ **(A)** and D_PC_ **(B)** for the phantom and the three patient cases. The white squares represent the stents of the legs overlapped with the main body stents, not included in the study.

Figure 10 shows the distance maps separating the barycenters of the simulated stents from the target stents, for the phantom case and the three patient cases. Each square represents a stent, with a colorbar related to error values. Overlapping stents between the main body and the limbs were not included because of segmentation issues. Table 3 reports the results shown in the different distance maps. For each dataset and for each criterion, the mean, standard deviation, maximum and minimum values over all stents of the corresponding case are presented. To summarize Table 3, for Dataset I, mean **D**_B_ = 1.65 mm and mean **D**_PC_ = 1.85 mm. For Dataset II, mean **D**_B_ = 1.35 mm and mean **D**_PC_ = 1.53 mm.

**Table 3:** Summary of results obtained for Dataset I and II: mean, standard deviation, maximum, and minimum values.

		**D**_**B**_ **(mm)**				**D**_****P*****C*****_ **(mm)**			
**Dataset**	**Description**	**Mean**	**Std**	**Max**	**Min**	**Mean**	**Std**	**Max**	**Min**
I	Phantom	1.65	0.80	2.95	0.48	1.85	0.89	3.73	1.02
II	Case 1	1.12	0.99	4.99	0.13	1.26	0.50	3.22	0.64
	Case 2	1.06	0.71	2.69	0.32	1.60	0.36	2.43	1.29
	Case 3	0.87	0.43	2.02	0.15	1.74	0.43	2.90	0.90

Criterion **D**_B_, similar to the criterion used to evaluate stent positioning, was used to measure the average distance between target and simulated fenestrations. The average distances were **D**_B_ = 0.41 mm for the right renal fenestration, **D**_B_ = 1.64 mm for mesenteric fenestration and **D**_B_ = 2.46 mm for the left renal fenestration.

## Discussion

In this study, we introduced a novel method for fast numerical simulations of FEVAR procedures and we performed a thorough validation analysis relying first on *in vitro* data obtained on a 3D printed phantom, and then on *in vivo* data obtained on 3 FEVAR patients. Motion of the stent-graft after the deployment was not considered here. Any motion that would occur during the procedure and that would be imaged by fluoroscopy could be traced by the model, but not post-surgery motions.

We first achieved a qualitative evaluation of the simulation accuracy. Globally, and in all cases, the shape of the simulated stent graft matched the target, inside, and outside the target image plane. There were no significant errors on the shape of the device. Considering the stents individually, the simulations based on both Datasets I and II (Figure 7) were close to the targets. Errors were difficult to identify visually, except for the stent of the phantom case located just above the bifurcation, which showed deployment errors in the lateral view (Figure 7). Since this stent was in the aneurysm sac, its deployment was not constrained by the aorta, which has a larger diameter than the deployed stent. Actually, the fabric prevented complete stent-graft deployment, but not in the simulation since fabric was not implemented. Therefore, the errors in the simulation were mostly related to the absence of the fabric (*ES fabric*).

Figures 8, 9 showed that the simulated fenestrations were well positioned in front of the targets. Although the fenestrations were not perfectly centered on the target, the overlap was sufficient to facilitate catheterization of the secondary arteries, which is the critical step for the success of FEVAR. We noticed few errors in the image plane, while the largest deviations appeared along the projection axis. This observation was consistent with the method, which used information in the image plane to simulate stents. Moreover, similar results were observed in 2D-3D registration methods, which reported higher out-of-plane errors compare to in-plane errors in single-view registration (41–43). Despite this error, the accuracy of the method in and out of the image plane seemed acceptable and sufficient to facilitate the visualization of the stent-graft.

We also achieved quantitative assessments of the simulation results. The simulation was qualified as *excellent* when **D** < 3 mm, *good* when **D** < 5 mm, and *insufficient* if **D** > 5 mm, according to a study evaluating fusion road map accuracy for EVAR (40). The observations made on the distance maps were consistent with the initial qualitative assessments. Concerning Dataset I, the phantom, Table 3 showed that the results were in average *excellent* both for **D**_B_ and **D**_PC_, although the maximum value achieved for **D**_PC_ was 3.73 mm, which ranked it in *good*. The method fully met clinical expectations, both in terms of stent positioning in the aorta and in terms of individual deployment. Maximum **D**_PC_ value were located above the bifurcation previously identified, located in the aneurysmal sac, while stents located in the healthy part of the normal artery had *excellent* results. This indicated that despite good stent positioning in the artery, deployment was not optimally simulated in the sac. This was consistent with the first observation made on the qualitative data, and seemed to confirm our initial hypothesis, i.e., simulation errors are related to *ES fabric* when the deployment is not constrained by the aortic wall. Concerning Dataset II, average results were *excellent*. Both on the distance maps and in Table 3, no value exceeded the 5 mm threshold, and the average **D**_B_ and **D**_PC_ were below 3 mm. The method met clinical accuracy expectations, despite the introduction of a new source of error. Interestingly, the maximum error **D**_B_=5 mm for the penultimate stent located at the extremity of the left leg of Case 1, was reduced to **D**_PC_=3.22 mm. This suggests that despite an approximate initial positioning in the artery, the deployment calculation improved the final accuracy of the simulation. These results are comparable with most pre-operative finite elements simulations (20, 21, 44, 45) and with shape instantiation methods (30, 31), although further comparison is limited by the diversity of measurements performed. Fast stenting methods obtain slightly better (46) or inferior results (26–28), but in most cases the aortic geometries were simpler, these methods being challenged by complex configurations.

Average precision obtained for Dataset II is better than Dataset I, regardless of the measurement (Table 3), although a new source of error, *ES stiffness*, has been introduced. This suggested that *ES stiffness* had a limited influence on the simulation results, which was consistent with our expectations. Indeed, the radial deployment force of the stents was small compared to the apparent stiffness of the artery walls. The improvement in the results compared to Dataset I could be explained by the aortic geometries. The phantom had a diameter larger than the patient's aneurysm. Our previous observation based on Dataset I results was that *ES fabric* is more significant when the deployment is not constrained by aorta wall, *i.e*. in the aneurysm sac. In agreement with this previous observation, *ES fabric* had only a limited contribution because the actual aneurysm diameters were lower than in the phantom. Globally, the results obtained with Dataset II were extremely promising and showed that *ES stiffness* was negligible.

It should be noted that the stents were all deployed individually to fully evaluate the method. In a clinical context, only the stents of interest would be simulated, for example stents near ostia and stents with fenestrations. The results concerning the positioning of the fenestrations were fully satisfying. The average distance **D**_B_ between target and simulated fenestrations was *excellent* for all the fenestrations. These results are comparable with those obtained from comprehensive finite element analyses predicting rotation in FEVAR, achieved in several hours (47).

The accuracy achieved here is comparable to that obtained by the 3D shape instantiation method based on the segmentation and identification of radiopaque markers (30, 31). The computation time of the latter is very short, much less than the total computation time of FMVSD. However, this method is limited by the size of the markers and would require new markers for improving accuracy. Other methods are based on simplified mechanical models and obtain similar precision results in a short time (46). However, these methods are challenged by the complex geometries, and do not use the valuable information from intraoperative imaging (26–28). Finally, methods based on pre-operative finite element analysis can simulate the deployment with better accuracy, but at the cost of high computational time and again ignoring valuable intraoperative information (44, 45). One study especially highlighted that a significant difference was observed between simulation and ground truth, due to unpredictable physician manipulation during stent graft positioning (19). Therefore, our approach offers a good compromise between computation time or accuracy.

Although the results were compatible with clinical expectations, this study suffers from some limitations. First, not enough cases were tested in order to allow meaningful statistical analyses. Moreover, the method could not be tested on a complete data set, i.e., including preoperative scans, postoperative scans and intraoperative images for the same case. Clinical intraoperative images were missing, which made it impossible to evaluate the method under clinical conditions. There were other potential sources of error other than those listed above, which were neglected here: errors in the segmentation of the aorta of the thrombus, errors in the projection matrix calibration, simplified mechanical behavior, and geometry of the stent-graft, errors induced by image processing.

The total computation time was about 6–7 min, mostly at Step 2. The total computation time is the time required for the simulations and does not include model generation (which can take up to 1 h). This time is slightly higher than the acceptable time limit agreed with the clinicians, which is 5 min. However, the computation time can be easily reduced in the future: in a clinical context, only a few stents could be simulated whereas all of them were simulated in the results reported here. Moreover, the current implementation did not consider optimal contact algorithms and convergence criteria. Finally, parallelization is easily feasible, allowing to simulate the deployment of the stents simultaneously in a very short time. After optimization and parallelization, the computation time should be compatible with a clinical workflow.

Surgical simulations have an increasingly important role to play, whether for training practitioners or to assist them during pre-operative planning and while performing surgery. The verification and validation of surgical assistance tools is of importance, since the information provided by the simulation may influence the choices made by the surgeon. The physician must have sufficient confidence in the tool to be able to make rapid decisions in the surgical context. However, the final decision will not rely entirely on an assistance tool, which is complementary by definition, and the clinician will support his decision with intraoperative imaging. Thus, although absolutely necessary, the verification and validation of such a method is not as critical as the validation of the implantable device itself. The verification and validation procedure followed in this article is relatively standard and included validation steps on phantom and clinical data. In order to validate a simulation method, the most direct approach is to compare the simulation result with the ground truth, obtained from phantoms, clinical data, or gold standards. In our case, we compared the geometries of the simulated stents with the geometries extracted from the phantom and the clinical data. The deployment procedure achieved in the aorta phantom allowed a total control of the different parameters of the procedure, at the detriment of realism. This validation approach is commonly used to evaluate simulation or registration methods. For example, a silicone mock aneurysm was used to validate the deployment simulation of a bifurcated stent graft (19), and aneurysms were 3D printed to validate a stent graft shape instantiation method in EVAR (30, 31). Complex dynamic physical model has also been used to evaluate a segmentation method to measure sent graft motion (48). The second part of the validation was performed on clinical data, which implied a lower control of the environment parameters compared to phantom simulations, but which had the advantage of being based on real data, replicating clinical settings and the associated uncertainties. In our case, validation was done using retrospective data. Validation on clinical data is widely used in the field of cardiovascular devices, especially in FEVAR, for example to predict fenestration rotation (47), iliac complications (49), or to validate numerical simulation of stent deployment (7, 8, 18, 21, 22, 25, 50). Other numerical methods have been validated by combining phantom and clinical data (11, 12, 29, 51).

A variation of these standard validation schemes, which is less frequently found in the literature, is the use of different datasets in order to isolate the different sources of error in the model. Such validation methods, based on the identification of different sources of error, have been proposed previously to evaluate accuracy of fusion road map for EVAR (40), image guided endoscopic cranial surgery (52), or orthopedic surgery (53). Besides allowing a global verification of the method, the use of different datasets with varying constraints and unknowns enables, to some extent, to test separately the errors related to the major simplifications of the model. For example, this allowed us to conclude that the errors related to the wall stiffness were negligible compared to the errors related to the fabric. Simplifications are inherent to simulations, and this is especially true for intraoperative simulations which must be performed in a relatively short time. The development of such validation method would be a convenient way to validate the numerical simulation while identifying the main sources of error, which could then be improved and optimized in future iterations of the algorithm. It would therefore seem appropriate to design a testing framework to perform experiments under various conditions to isolate and evaluate the various sources of error identified previously. In our case, the use of phantom but also the access to pre- and post-operative data from clinical cases enabled to set up this framework at a reduced scale, which is sufficient for a proof of concept. Although reduced, this first validation allowed to identify the fabric simulation as the major source of error. Thus, while waiting for a more complete validation of the method, essential before considering its use in a clinical context, an optimization work on this feature could be initiated.

We presented here a new method for the simulation of stent-graft deployment during (F)EVAR. The performance of the method was quantified, and the hypothesis that were considered most likely to induce simulation errors were evaluated. Overall, all errors for both stent and fenestration positioning were less than 5 mm, making this method compatible with clinical expectations. More specifically, the errors related to fenestration positioning were less than 3 mm, which is excellent according to the classification used in the literature. Thus, the FMVSD method, based on a single intraoperative image, could achieve an accuracy compatible with clinical expectations with limited calculation time. Although requiring further validation, this method is therefore very promising and can be adapted and coupled with hardware currently used in surgical rooms in order to assist practitioners and help them to reduce the number of Xray acquisitions needed during FEVAR interventions.

## Data Availability Statement

The original contributions presented in the study are included in the article/supplementary material, further inquiries can be directed to the corresponding author/s.

## Ethics Statement

The studies involving human participants were reviewed and approved by Comité de Protection de la Personne, CHU Saint-Etienne. The patients/participants provided their written informed consent to participate in this study.

## Author Contributions

SG, J-NA, BP, and SA: conceptualization and supervision. AP: formal analysis and writing—original draft. BP and AP: investigation. AP, SG, J-NA, BP, and SA: methodology. SG and SA: project administration and resources. J-NA and BP: visualization. BP and SA: writing—review and editing. All authors contributed to the article and approved the submitted version.

## Conflict of Interest

The authors declare that the research was conducted in the absence of any commercial or financial relationships that could be construed as a potential conflict of interest.
